# Influence of sex and advanced age on outcomes after endovascular aneurysm repair: a 13-year single-center retrospective cohort study

**DOI:** 10.3389/fcvm.2025.1687919

**Published:** 2026-01-08

**Authors:** Ersin Ozer, Kaptaniderya Tayfur, Melih Urkmez, Ferhat Borulu

**Affiliations:** 1 Department of Cardiovascular Surgery, Ordu University Education and Research Hospital, Ordu, Türkiye; 2 Department of Cardiovascular Surgery, Faculty of Medicine, Ordu University, Ordu, Türkiye

**Keywords:** abdominal aortic aneurysm, endovascular procedures, age factors, sex characteristics, treatment outcome, endoleak

## Abstract

**Introduction:**

Sex and advanced age may influence anatomical complexity and clinical outcomes after endovascular abdominal aortic aneurysm repair (EVAR). This study evaluated the impact of sex and age ≥ 80 years on peri-operative and long-term outcomes following EVAR.

**Methods:**

We conducted a 13-year, single-center retrospective cohort study of 512 patients (311 men, 201 women) who underwent EVAR for infrarenal abdominal aortic aneurysm between 2010 and 2023. Patients were stratified by sex and by age (<80 vs. ≥80 years). The primary endpoint was long-term all-cause mortality. Secondary endpoints included perioperative outcomes, Type I endoleak incidence, and reintervention rates. Mortality predictors were assessed using Cox proportional hazards regression. Median follow-up time was 95 months.

**Results:**

Women and octogenarians had significantly larger aneurysm diameters and greater aortic neck angulation (both *p* < 0.001). Type I endoleak incidence was significantly higher in women (26.4% vs. 11.9%, *p* < 0.001) and in patients ≥80 years (32.2% vs. 11.4%, *p* < 0.001). Cox regression demonstrated that age ≥80 years increased mortality risk 2.43-fold [hazard ratio (HR) = 2.430; 95% confidence interval: 1.430–4.127; *p* = 0.001], whereas sex was not an independent predictor of mortality (*p* = 0.185).

**Discussion:**

Octogenarians exhibited markedly higher mortality risk, and women presented with more challenging vascular anatomy and a higher rate of Type I endoleaks. These findings are consistent with reported anatomical and outcome disparities in high-risk AAA populations.

**Conclusion:**

These results emphasize that tailored pre-operative planning, device selection, and long-term follow-up strategies may optimise outcomes in elderly and female patients and align with contemporary vascular surgery guidelines.

## 1 Introduction

Abdominal aortic aneurysm (AAA) is an irreversible, progressive, and degenerative vascular pathology characterized by enlargement to at least twice the expected transverse diameter for the individual's age and body surface area in any segment of the infradiaphragmatic aorta (1, 2). AAA is one of the leading causes of cardiovascular mortality in the elderly (3). Its prevalence of AAA is significantly increasing, particularly in men aged ≥65 years, with rising diagnosis rates also reported in women (4). Endovascular treatment, a minimally invasive method, has emerged as an alternative to open surgical repair for AAA (5). Age, female sex, complex aneurysm anatomy, and cardiac, pulmonary, and renal comorbidities are associated with higher rates of postoperative complications following AAA treatment (6). Endovascular aneurysm repair (EVAR) has become an essential option for patients with high perioperative risk because its associated morbidity and mortality are lower than those of conventional treatments (7, 8).

However, evidence suggests that EVAR offers greater benefit in men. Several studies have reported that differences in female vascular anatomy are associated with increased perioperative mortality, higher complication rates, and a greater need for secondary intervention (9–17). In this context, contemporary guidelines, such as the European Society for Vascular Surgery (ESVS) 2024 Clinical Practice Guidelines, emphasize nuanced patient selection and long-term surveillance tailored to demographic factors, including sex and advanced age (18).

Despite these findings, few large, contemporary single-center studies have comprehensively evaluated the combined influence of female sex and advanced age (≥80 years) on EVAR outcomes over long-term follow-up. Most available literature focuses on only one demographic factor or relies on database analyses that lack detailed anatomical parameters. Importantly, there is a gap in understanding how the unique anatomical challenges in women (e.g., smaller vessels, greater angulation) interact with the systemic and procedural complexities of octogenarians when both factors coexist. This study aims to address this gap by evaluating the effects of sex and age on outcomes in patients who underwent EVAR for AAA at a single institution over 13 years. The findings may help refine treatment strategies for these patient groups.

## 2 Materials and methods

We identified 512 patients who underwent EVAR for infrarenal AAA between 2010 and 2023. Of these, 311 were male (60.7%), 201 were female (39.3%), and 152 (29.6%) were aged ≥80 years. The study was approved by the Ordu University Clinical Research Ethics Committee (decision no. 2023/364; 27/12/2023). The cohort included patients with intact infrarenal AAA. Patients with ruptured AAAs were excluded to avoid confounding outcome analyses and to focus on elective cases.

Patients were evaluated for EVAR candidacy based on anatomical criteria determined through preoperative contrast-enhanced computed tomography (CT) and, when indicated, angiography. Anatomical inclusion was based on the feasibility of standard EVAR device placement in accordance with contemporary vascular surgery guidelines, rather than strict predefined anatomical thresholds. Patients who underwent concurrent visceral interventions or thoracic endovascular aortic repair were excluded.

The choice of endograft devices was guided by evolving surgical preferences and patient anatomy. In total, 204 patients received a Gore Excluder device, and 308 received a Medtronic Endurant device. Radiological images and peri- and post-procedural data were recorded prospectively in an established database and analyzed retrospectively. Informed consent was obtained from all patients before the procedure. This prospective data collection provides a robust foundation and minimizes the limitations associated with purely retrospective data retrieval.

### 2.1 Endovascular repair technique

EVAR was performed in the angiography unit under general or regional anesthesia in a sterile operating environment. Standard bilateral femoral artery exposure via surgical cutdown was used, and 5,000 units of heparin were administered in accordance with standard device instructions for use.

### 2.2 Follow-up

After discharge, patients were followed up at 1, 6, and 12 months, and annually thereafter. Follow-up included CT angiography or abdominal color Doppler ultrasonography at each visit to ensure the reliability and validity of the longitudinal assessment. The median follow-up duration was 95 months (range: 2–13 years), providing confidence in the study's comprehensiveness and the reliability of its conclusions.

### 2.3 Study endpoints

The primary endpoint of this study was long-term all-cause mortality. Secondary endpoints included the following:

▪ Perioperative outcomes: operation time and hospital length of stay
▪ Aneurysm morphology: aneurysm diameter, aortic neck diameter, neck length, and aortic neck angulation
▪ Complication rates: incidence of Type I, II, and III endoleaks
▪ Long-term outcomes: need for secondary reinterventions (e.g., balloon angioplasty, bypass, cuff placement)

### 2.4 Statistical analysis

Before analysis, the dataset was reviewed to identify and correct missing values, errors, and outliers. Descriptive analysis included frequencies and percentages for categorical variables and measures of central tendency (mean, median) and variability (standard deviation, interquartile range) for numerical data. The Kolmogorov–Smirnov and Shapiro–Wilk tests were employed to evaluate the normal distribution of the data. Skewness and kurtosis (and their z-scores) were also evaluated. Levene's test was used to verify the homogeneity of variances.

Comparisons between two independent groups were performed using the independent-samples *t*-test or Mann–Whitney *U*-test based on parametric assumptions. Associations between categorical variables were examined using the Chi-square test. Cox proportional hazards regression analysis was performed to assess the effects of demographic characteristics and treatment duration on mortality outcomes, with sex, age group, and treatment duration included as predictor variables. Hazard ratios (HRs) and 95% confidence intervals (CIs) were calculated. Variable selection for the multivariate Cox model was guided by the need to maintain a robust model and avoid multicollinearity among anatomical variables (e.g., neck angulation, aneurysm diameter) as well as overfitting due to limited events. This approach improves the generalizability and stability of our main findings. Statistical significance was set at α = 0.05. Analyses were conducted using R (version 4.4.1), IBM SPSS (version 26), and MedCalc (version 21).

## 3 Results

In the study population, smoking prevalence was significantly higher among males (*p* < 0.001) and similarly higher in patients under 80 years of age (*p* = 0.008). All cases presented with AAA, and 90% underwent spinal anesthesia. Hypertension (46.8%) was the most prevalent comorbidity, followed by diabetes mellitus (17.9%) and chronic obstructive pulmonary disease (15.1%). Comorbidity distribution did not differ significantly across sex or age groups (*p* > 0.05). Demographic characteristics are presented in Tables 1, 2.

**Table 1:** Demographic characteristics of the patients (1).

Variable		Gender							Age							Total (*N*)	Column (%)
		Male			Female			*p*	<80			>=80			*p*		
		*N*	Row	Column	N	Row	Column		*N*	Row	Column	*N*	Row	Column			
Smoke	Yes	155	85.2	49.8	27	14.8	13.4	**<0.001**	141	77.5	39.2	41	22.5	27.0	**0.008**	182	35.5
	No	156	47.3	50.2	174	52.7	86.6		219	66.4	60.8	111	33.6	73.0		330	64.5
HT	Yes	170	60.7	54.7	110	39.3	54.7	0.989	202	72.1	56.1	78	27.9	51.3	0.319	280	54.7
	No	141	60.8	45.3	91	39.2	45.3		158	68.1	43.9	74	31.9	48.7		232	45.3
DM	Yes	78	61.9	25.1	48	38.1	23.9	0.758	87	69.0	24.2	39	31.0	25.7	0.720	126	24.6
	No	233	60.4	74.9	153	39.6	76.1		273	70.7	75.8	113	29.3	74.3		386	75.4
COPD	Yes	51	58.6	16.4	36	41.4	17.9	0.656	54	62.1	15.0	33	37.9	21.7	0.065	87	17.0
	No	260	61.2	83.6	165	38.8	82.1		306	72.0	85.0	119	28.0	78.3		425	83.0
CAD	Yes	71	64.0	22.8	40	36.0	19.9	0.432	72	64.9	20.0	39	35.1	25.7	0.156	111	21.7
	No	240	59.9	77.2	161	40.1	80.1		288	71.8	80.0	113	28.2	74.3		401	78.3
CAS	No	311	60.7	100.0	201	39.3	100.0	NA	360	70.3	100.0	152	29.7	100.0	NA	512	100.0
PAD	Yes	28	80.0	9.0	7	20.0	3.5	**0.016**	25	71.4	6.9	10	28.6	6.6	0.881	35	6.8
	No	283	59.3	91.0	194	40.7	96.5		335	70.2	93.1	142	29.8	93.4		477	93.2
CKD	Yes	11	73.3	3.5	4	26.7	2.0	0.311	13	86.7	3.6	2	13.3	1.3	0.159	15	2.9
	No	300	60.4	96.5	197	39.6	98.0		347	69.8	96.4	150	30.2	98.7		497	97.1
Malignancy	No	311	60.7	100.0	201	39.3	100.0	NA	360	70.3	100.0	152	29.7	100.0	NA	512	100.0
CD	Yes	9	52.9	2.9	8	47.1	4.0	0.503	13	76.5	3.6	4	23.5	2.6	0.572	17	3.3
	No	302	61.0	97.1	193	39.0	96.0		347	70.1	96.4	148	29.9	97.4		495	96.7
Total		311	60.7	100.0	201	39.3	100.0		360	70.3	100.0	152	29.7	100.0		512	100.0

HT, hypertension; DM, diabetes mellitus; COPD, chronic obstructive pulmonary disease; CAD, coronary artery disease; CAS, carotid artery stenosis; PAD, peripheral artery disease; CKD, chronic kidney disease; CD, cerebrovascular disease; NA, not applicable.

Bold values indicate statistically significant results (*p* < 0.05).

**Table 2:** Demographic characteristics of the patients (2).

Variable		Gender						*p*	Age						*p*	Total
		Male			Female				<80			>=80				
		*N*	Row	Column	*N*	Row	Column		*N*	Row	Column	*N*	Row	Column		
Type of Anesthesia	General	30	58.8	9.6	21	41.2	10.4	0.767	36	70.6	10.0	15	29.4	9.9	0.964	51
	Spinal	281	61.0	90.4	180	39.0	89.6		324	70.3	90.0	137	29.7	90.1		461
Iliac Angle	Yes	90	59.2	28.9	62	40.8	30.8	0.645	78	51.3	21.7	74	48.7	48.7	**<0.001**	152
	No	221	61.4	71.1	139	38.6	69.2		282	78.3	78.3	78	21.7	51.3		360
Endoleak	No	264	65.8	84.9	137	34.2	68.2	**<0.001**	304	75.8	84.4	97	24.2	63.8	**<0.001**	401
	Type 1	37	41.1	11.9	53	58.9	26.4		41	45.6	11.4	49	54.4	32.2		90
	Type 2	8	53.3	2.6	7	46.7	3.5		12	80.0	3.3	3	20.0	2.0		15
	Type 3	2	33.3	0.6	4	66.7	2.0		3	50.0	0.8	3	50.0	2.0		6
Secondary Intervention	No	261	64.6	83.9	143	35.4	71.1	N.A	306	75.7	85.0	98	24.3	64.5	N.A	404
	Balloon angioplasty	13	48.1	4.2	14	51.9	7.0		13	48.1	3.6	14	51.9	9.2		27
	Bypass	14	77.8	4.5	4	22.2	2.0		13	72.2	3.6	5	27.8	3.3		18
	Bypass+ balloon angioplasty	9	56.3	2.9	7	43.8	3.5		9	56.3	2.5	7	43.8	4.6		16
	Cuff	7	21.9	2.3	25	78.1	12.4		12	37.5	3.3	20	62.5	13.2		32
	Cuff+bypass	0	0	0	1	100.0	.5		1	100.0	0.3	0	0	0		1
	Additional stent	2	33.3	0.6	4	66.7	2.0		3	50.0	0.8	3	50.0	2.0		6
	PTCA	2	66.7	0.6	1	33.3	0.5		1	33.3	0.3	2	66.7	1.3		3
	PTCA+ balloon angioplasty	2	50.0	0.6	2	50.0	1		1	25.0	0.3	3	75.0	2.0		4
	PTCA+cuff	1	100.0	0.3	0	0	0		1	100.0	0.3	0	0	0		1

PTCA, percutaneous transluminal coronary angioplasty; N.A, not applicable.

Bold values indicate statistically significant results (*p* < 0.05).

A gender-based assessment revealed that women had significantly larger aneurysm diameters (67.88 ± 10.20 mm vs. 66.11 ± 8.90 mm, *p* = 0.039) and greater aortic neck angulation (56.98 ± 15.74° vs. 43.68 ± 14.76°, *p* < 0.001) than men. When evaluated by age, patients ≥80 years had significantly larger aneurysm diameters (72.95 ± 11.73 mm vs. 64.21 ± 6.84 mm, *p* < 0.001) and greater neck angulation (56.53 ± 17.97° vs. 45.67 ± 14.68°, *p* < 0.001). These comparisons are presented in Table 3.

**Table 3:** The comparison results of clinical measurements across gender levels.

Variable	Gender											Age										
	Male					Female					*p*	<80					>=80					*p*
	Mean	SD	Median	IQR		Mean	SD	Median	IQR			Mean	SD	Median	IQR		Mean	SD	Median	IQR		
Aneurysm diameter	66.11	8.90	64.52	59.84	70.36	67.88	10.20	66.00	60.25	71.44	**0.039**	64.21	6.84	63.18	59.35	68.44	72.95	11.73	70.59	64.10	78.46	**<0.001**
Neck distance	2.45	0.69	2.40	2.00	2.80	2.40	0.59	2.30	2.10	2.60	0.370	2.54	0.66	2.50	2.10	3.00	2.17	0.55	2.20	1.85	2.50	**<0.001**
Aortic neck diameter	25.86	3.71	26.32	23.78	28.55	26.85	3.27	26.38	24.55	29.31	**0.025**	25.79	3.38	25.69	23.94	28.31	27.33	3.80	28.35	25.23	30.22	**<0.001**
Aortic neck angle	43.68	14.76	43.00	33.00	53.00	56.98	15.74	58.00	45.00	66.00	**<0.001**	45.67	14.68	45.00	35.00	56.00	56.53	17.97	56.00	45.00	69.00	**<0.001**
Right iliac artery diameter	19.21	6.29	17.66	15.31	21.15	20.41	7.23	19.22	16.32	22.30	**0.046**	18.94	6.27	17.39	15.31	20.32	21.44	7.33	19.65	17.41	23.43	**<0.001**
Left iliac artery diameter	19.96	6.16	18.52	16.00	22.22	21.07	6.40	19.65	16.71	22.54	**0.014**	19.46	5.41	18.45	16.00	21.47	22.62	7.52	21.31	16.93	24.81	**<0.001**

SD, standard deviation; IQR, interquartile range.

Bold values indicate statistically significant results (*p* < 0.05).

Operation duration was longer in women (52.44 ± 11.30 min vs. 49.81 ± 9.72 min, *p* = 0.005) and in patients ≥80 years (54.57 ± 11.07 min vs. 49.27 ± 9.75 min, *p* < 0.001). Length of hospital stay was also significantly longer in both women and patients ≥80 years (*p* < 0.001). Time-related outcomes are presented in Table 4.

**Table 4:** The comparison of time measurements at gender levels.

Variable	Gender											Age										
	Male					Female					*p*	<80					>=80					*p*
	Mean	SD	Median	IQR		Mean	SD	Median	IQR			Mean	SD	Median	IQR		Mean	SD	Median	IQR		
Follow-up time	90.95	37.91	95	69	122	94.50	32.51	96	76	120	0.260	93.84	35.95	96.5	72	122	88.80	35.62	88.5	72	116.5	0.115
Operation time	49.81	9.72	50	45	55	52.44	11.30	50	45	60	**0.005**	49.27	9.75	45	45	55	54.57	11.07	55	45	62.5	**<0.001**
Hospitalisation duration	1.29	0.50	1	1	2	1.54	0.63	1	1	2	**<0.001**	1.26	0.49	1	1	1	1.68	0.62	2	1	2	**<0.001**

SD, standard deviation; IQR, interquartile range.

Bold values indicate statistically significant results (*p* < 0.05).

Type I endoleak rates were higher in women (26.4%) compared to men (11.9%) and in patients ≥80 years (32.2%) compared to younger patients (11.4%) (*p* < 0.001). These rates include both intraoperative and late Type I endoleaks. Iliac angulation was notably more common in patients ≥80 years (48.7% vs. 21.7%, *p* < 0.001). Associations with iliac angulation are summarized in Table 5.

**Table 5:** The results of the associations between iliac angulation gender and age groups.

Variable		Male			Female				< 80 years of age			>= 80 years of age				
		*N*	Row (%)	Column (%)	*N*	Row (%)	Column (%)		*N*	Row (%)	Column (%)	*N*	Row (%)	Column (%)		
Iliac angle	Yes	90	59.2	28.9	62	40.8	30.8	0.645	78	51.3	21.7	74	48.7	48.7	**<0.001**	152
	No	221	61.4	71.1	139	38.6	69.2		282	78.3	78.3	78	21.7	51.3		360
Graft type	Polyester	181	59.2	58.2	125	40.8	62.2	0.369	208	68.0	57.8	98	32.0	64.5	0.158	306
	PTFE	130	63.1	41.8	76	36.9	37.8		152	73.8	42.2	54	26.2	35.5		206
Graft leg thrombosis		Mean		SD	Mean		SD	0.359	Mean		SD	Mean		SD	0.626	152
		58.43		39.79	47.67		28.19		56.88		41.49	51.30		27.01		
Graft type	Polyester	61.00		42.51	41.67		22.26	0.082	56.00		42.71	52.29		29.84	0.778	181
	PTFE	43.00		5.72	71.67		41.93	0.358	61.75		39.23	46.67		2.89	0.545	130

PTFE, politetrafloroetilen; SD, standard deviation.

Bold values indicate statistically significant results (*p* < 0.05).

The comparison results of ex-status and ex-period measurements are presented in Table 6. The median follow-up period for the entire cohort was 95 months (range: 2–13 years). Of the 512 patients, 454 (88.7%) were censored (alive or lost to follow-up at the end of the study period). Cox regression analysis revealed that age ≥80 years increased mortality risk by 2.43-fold (HR = 2.430, 95% CI: 1.430–4.127, *p* = 0.001), whereas sex was not independently associated with mortality (HR = 1.426, 95% CI: 0.843–2.411, *p* = 0.185). Cox regression results are presented in Table 7.

**Table 6:** The comparison results of ex status and ex period measurements across gender and age groups.

Variable		Gender						*p*	Age						*p*	Total
		Male			Female				<80			>=80				
		*N*	Row	Column	*N*	Row	Column		*N*	Row	Column	*N*	Row	Column		
E×Cause	No	283	62.3	91.0	171	37.7	85	NA	330	72.7	91.7	124	27.3	81.5	NA	454
	MI	11	47.8	3.5	12	52.2	6.0		15	65.2	4.2	8	34.8	5.3		23
	CVD	11	42.3	3.5	15	57.7	7.5		8	30.8	2.2	18	69.2	11.8		26
	Cancer	5	71.4	1.7	2	28.6	1.0		6	85.7	1.7	1	14.3	0.7		7
	Rupture	1	50.0	0.3	1	50.0	0.5		1	50.0	0.3	1	50.0	0.7		2
	Total	311	60.7	100.0	201	39.3	100.0		360	70.3	100.0	152	29.7	100.0		512
Ex (Year)		*N*	Mean	SD	*N*	Mean	SD	*p*	*N*	Mean	SD	*N*	Mean	SD	*p*	58
		28	8.46	2.91	30	11.73	10.49	0.110	30	9.80	7.07	28	10.54	8.85	0.727	

MI, myocardial infarction; CVD, cerebrovascular disease; SD, standard deviation.

**Table 7:** The results of Cox survival regression analysis based on gender and age groups.

Variables in the equation									Overall model performance		
Variable	B	SE	Wald	df	Sig.	Exp(B)	95.0% CI for Exp(B)		-2LL	Chi-square	*p*
							Lower	Upper			
Age (>=80)	0.888	0.270	10.786	1	0.001	2.430	1.430	4.127	570.069	15.716	**<0.001**
Gender (female)	0.355	0.268	1.755	1	0.185	1.426	0.843	2.411			

B, coefficients; SE, standard error; df, degrees of freedom; Sig., significance; Exp(B), hazard ratios; CL, confidence interval; -2LL, log-likelihood ratio.

Bold values indicate statistically significant results (*p* < 0.05).

The primary causes of long-term mortality were myocardial infarction and cerebrovascular events. There was no 30-day mortality in this elective cohort. The overall secondary reintervention rate was 18.6% (*n* = 95), and the most common procedures were cuff placement (*n* = 32) and balloon angioplasty (*n* = 27).

**Figure 1:**
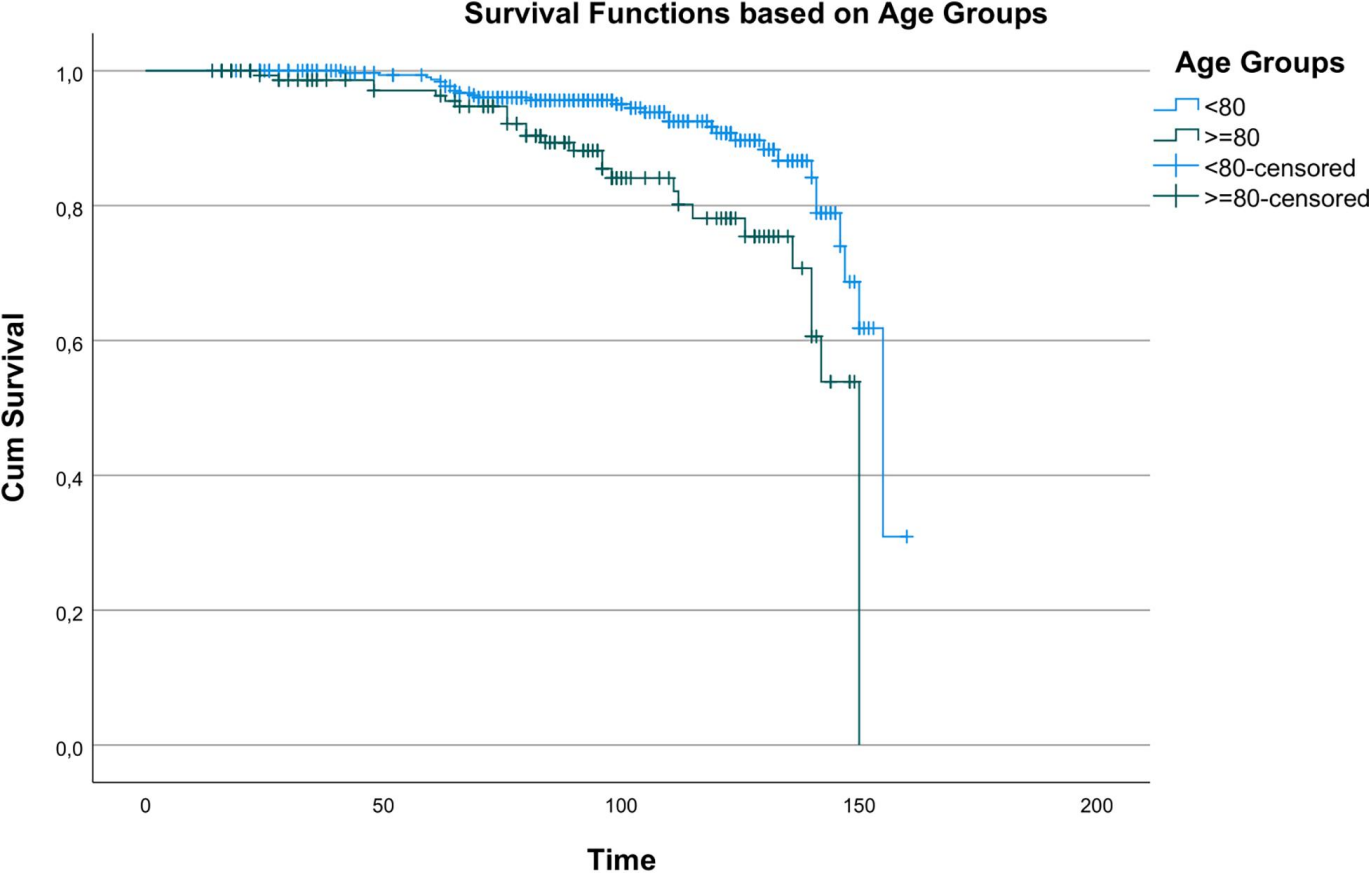
Kaplan–Meier survival curves showing overall survival according to age groups (<80 vs. ≥80 years).

**Figure 2:**
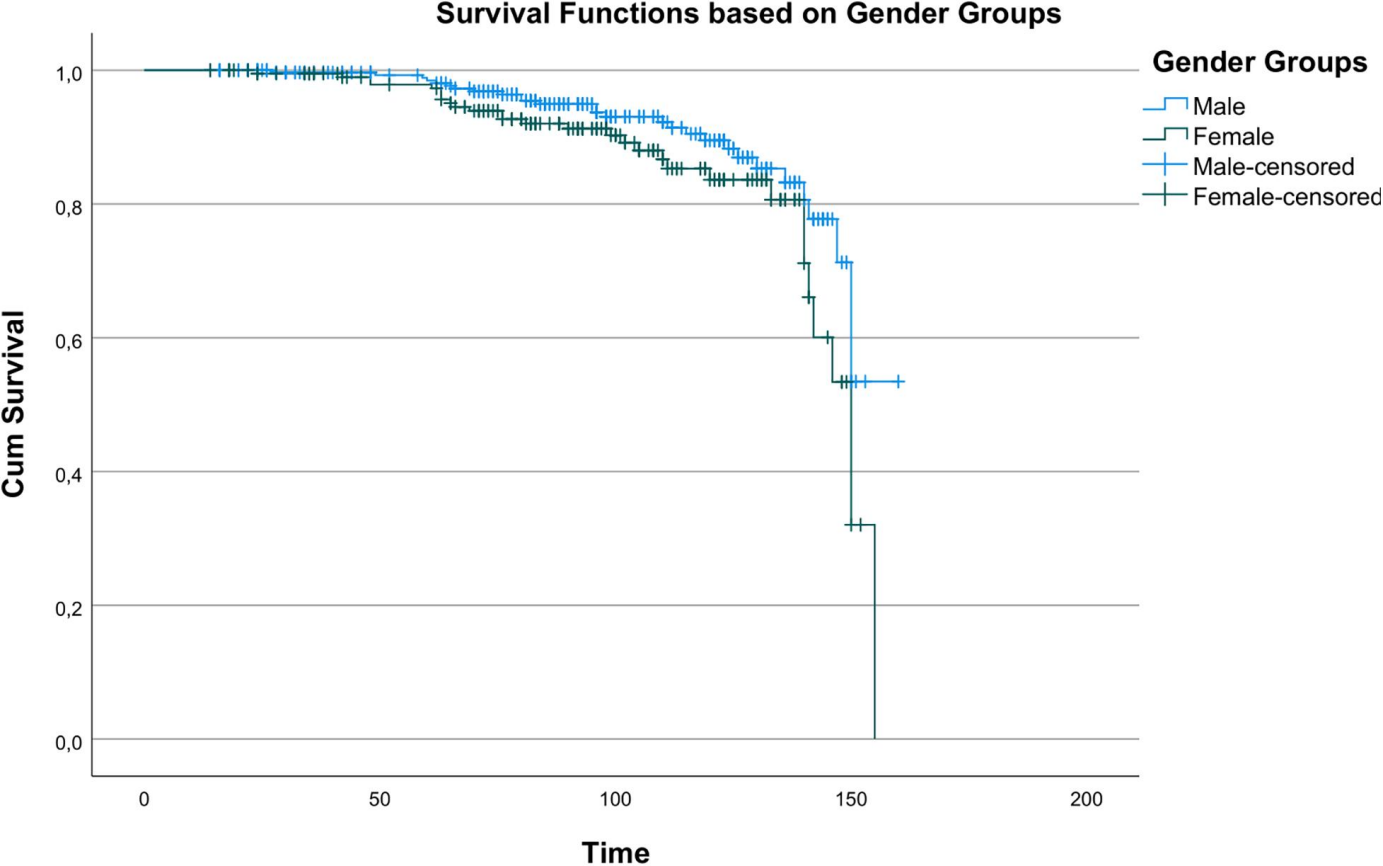
Kaplan–Meier survival curves showing overall survival according to gender groups (male vs. Female).

Kaplan–Meier analysis revealed statistically significant differences in survival between age groups (*χ*^2^ = 14.196, *p* < 0.001), indicating age as a key determinant of long-term survival. Survival curves by age are shown in Figure 1. A statistically significant but less pronounced survival difference was observed between men and women (*χ*^2^ = 3.890, *p* = 0.049), as shown in Figure 2.

## 4 Discussion

Many studies have demonstrated a strong association between smoking and AAA; smoking increases the incidence and prevalence of AAA as well as the risk of rupture (19). In our study cohort, smoking prevalence was higher in men and in patients under 80 years. Lottman et al. similarly reported that smokers tend to be younger, although they identified no sex-based differences, which is contrary to our finding (20).

In our study, 90% of patients underwent spinal anesthesia; no differences between sex and age groups were observed. By contrast, Cheng et al. reported greater use of general anesthesia (65.3%), followed by spinal anesthesia (13.5%) and local and monitored anesthesia care (14.2%). They observed a significant difference between local/monitored and general anesthesia regarding hospital stay and operative time, but no significant difference in the effect of anesthesia type on mortality and morbidity (21). Current ESVS guidelines offer a weak recommendation favoring locoregional anesthesia over general anesthesia in elective EVAR (18).

Previous studies have shown differences across sex in comorbidities among patients undergoing AAA repair. Nevidomskyte et al. reported that women had a lower prevalence of coronary artery disease but were more likely to have COPD compared to men (22). Our cohort showed similar distributions of comorbidities across sex and age groups: hypertension (46.8%), diabetes mellitus (17.9%), and COPD (15.1%).

Another notable finding in our study was that larger iliac artery diameters were observed in women and in patients over 80 years old. This contrasts with the findings of Tran et al., who reported that, despite similar body mass index (BMI) values between sexes, women had significantly smaller luminal adventitial diameters in the common iliac, external iliac, and common femoral arteries. Their study concluded that women generally have a considerably smaller iliofemoral arterial system than men (23).

We also observed that women exhibited larger aneurysm diameters and greater aortic neck angulation. This finding aligns partly with Ayo et al., who reported greater aortic neck angulation in women but no significant difference in aneurysm diameter (24). Interestingly, our female patients presented with larger aneurysm diameters (67.88 ± 10.20 mm vs. 66.11 ± 8.90 mm, *p* = 0.039). This finding contrasts with much of the Western literature, which typically reports smaller aortic dimensions in women. We attribute this difference to delayed diagnosis and lower participation in screening among women, which may result in more advanced progression of aneurysm at the time of EVAR.

Similarly, our elderly cohort showed greater aneurysm diameter and neck angulation, which is consistent with findings by Locham et al. (25). Donnel et al. observed that increasing age, larger neck diameter, shorter neck length, and greater angulation were associated with higher rates of Type I endoleaks (26). Our higher Type I endoleak rates are attributable to the challenging anatomies of our cohort (characterized by short neck length and high angulation) as well as the extended follow-up duration, which enables detection of late-onset endoleaks related to neck dilation or device migration.

Recent literature continues to highlight gender-related disparities in EVAR outcomes. A prospective, single-center study by Soares et al. found higher perioperative mortality rates in women undergoing aortoiliac aneurysm repair (27). This observation aligns with our findings about increased procedural difficulty for women, evidenced by their significantly higher Type I endoleak rates and greater anatomical complexity. While our Kaplan–Meier analysis showed a somewhat significant sex-based survival difference (*p* = 0.049), sex did not remain an independent predictor of mortality in the multivariate Cox regression model. This suggests that advanced age accounts for much of the observed disparity, which is consistent with other registry data (27).

In our study, myocardial infarction and cerebrovascular events were found to be prominent causes of mortality. While age ≥80 years showed a significantly higher risk of death (two-fold increase), gender had no significant effect on the mortality rate. This observation is consistent with established evidence of the association of AAA with myocardial infarction and claudication in men aged over 65 years and with cerebrovascular events in women (28–30). Despite some reports suggesting higher mortality in women (13% higher) (31), our analysis did not identify sex as an independent predictor. A recent prospective cohort study by Soares et al. similarly reported a significantly higher perioperative mortality rate in octogenarians (10% vs. 4%, *p* = 0.036). This confirms our Cox regression finding, which identified age ≥ 80 as an independent factor increasing mortality risk by 2.43-fold. However, in contrast to our Kaplan–Meier analysis, which showed a significant reduction in long-term survival for the elderly (*p* < 0.001), the study found that survival rates and freedom from reintervention at 1,080 days were statistically similar between octogenarians and non-octogenarians. This difference may be attributed to the shorter follow-up duration (1,080 days vs. our 13-year range) or differences in patient selection. This reinforces the need for continuous, long-term surveillance data (32).

We observed a divergence between the Kaplan–Meier analysis, which indicated a statistically significant difference in survival based on sex (*p* = 0.049), and the multivariate Cox regression model, which revealed no independent effect of sex on mortality (*p* = 0.185). This discrepancy suggests that the apparent survival disadvantage observed for women in the univariate analysis is likely not due to sex itself, but rather to its association with strong confounding factors, particularly advanced age, which serves as a powerful independent predictor of mortality in our multivariate model.

These findings underscore the importance of comprehensive anatomical evaluation and appropriate selection of stent-grafts before EVAR. We advocate for surgeons to implement more rigorous postoperative follow-up and early intervention strategies for this patient demographic. Our study has limitations. First, the retrospective design and observational nature of the survey introduce inherent limitations that may impact the findings, such as potential selection bias and reliance on the available data. We also recognize the absence of BMI values in our analysis and that vascular diameters were not categorized separately as luminal and adventitial, which we consider shortcomings of our study. Given that gender and age groupings influenced the study's integrity, we believe it would be beneficial to investigate gender and age parameters in separate studies. Furthermore, we may not have fully captured the long-term effects and complications associated with EVAR in patients with severe neck angulation.

Additionally, the use of different endograft devices for EVAR introduces a potential source of variability in our results. The utilization of endografts was not standardized throughout the study period; 204 patients were treated with the Gore Excluder device, while 308 patients received the Medtronic Endurant device. Finally, it is important to note that the follow-up period for the study varied from 2 to 13 years. Extending the follow-up duration may yield more comprehensive results.

## 5 Conclusion

Our study reaffirms the importance of demographic factors such as age and sex in the treatment of endovascular aneurysms. We observed that an increased aneurysm diameter, a widened aortic neck angle, and higher endoleak rates are prevalent in women and older age groups, which contribute to greater technical difficulties and postoperative risks for these populations. While our findings indicate that mortality is age-dependent, we did not observe a significant effect of sex. Therefore, tailoring treatment approaches to each patient's unique clinical and anatomical characteristics is essential for improving clinical outcomes and ensuring long-term survival.

## Data Availability

The raw data supporting the conclusions of this article will be made available by the authors, without undue reservation.
